# Osteoradionecrosis of the cervical spine presenting with quadriplegia in a patient previously treated with radiotherapy for laryngeal cancer: a case report

**DOI:** 10.4076/1752-1947-3-7262

**Published:** 2009-06-10

**Authors:** Frederik Carl van Wyk, Manu-priya Sharma, Robert Tranter

**Affiliations:** 1 Department of Otolaryngology and Head and Neck Surgery, Brighton and Sussex University Hospitals NHS Trust, Eastern Road, Brighton, UK

## Abstract

**Introduction:**

Osteoradionecrosis of the mandible and temporal bones has been extensively reported in literature, but cases of avascular necrosis of the cervical spine following radiotherapy to the larynx appear to be extremely rare. A review of the English language literature has shown only one other case where radiotherapy treatment of a laryngeal carcinoma has resulted in osteoradionecrosis of the cervical spine.

**Case presentation:**

We present the case of a 65 year old male patient who suffered from osteoradionecrosis of the cervical spine 20 years after radiotherapy treatment for a T1aN0M0 laryngeal carcinoma resulting in quadriplegia.

**Conclusions:**

Radiotherapy carries a long-term risk of complications, including osteoradionecrosis which may present 20 years later with significant implications.

## Introduction

Osteoradionecrosis is defined as necrosis of the bone following irradiation [1]. It was first described by Regaud in 1922 [2]. Historically, it was thought to have been the consequence of radiation, trauma and infection: radiation producing damage, ensuing trauma allowing entry of bacteria with subsequent infection [3]. The authors present a case of osteoradionecrosis of the cervical spine leading to quadriplegia following radiotherapy for laryngeal carcinoma.

## Case presentation

In August 1982, a 40-year-old man presented with hoarseness of 8 months duration. He was an ex-smoker.

Examination with indirect laryngoscopy revealed a polypoidal lesion on the middle third of a mobile right vocal cord. Clinical examination revealed no lymphadenopathy. Biopsy confirmed the presence of a squamous carcinoma.

Treatment consisted of stripping of the vocal cord and a full course of postoperative external beam radiotherapy for 30 treatments in 43 days - right and left lateral wedged fields totalling 6600cGy to the larynx - dose per fraction was 220cGy. Recovery was uneventful, apart from intermittent hoarseness.

**Figure 1:**
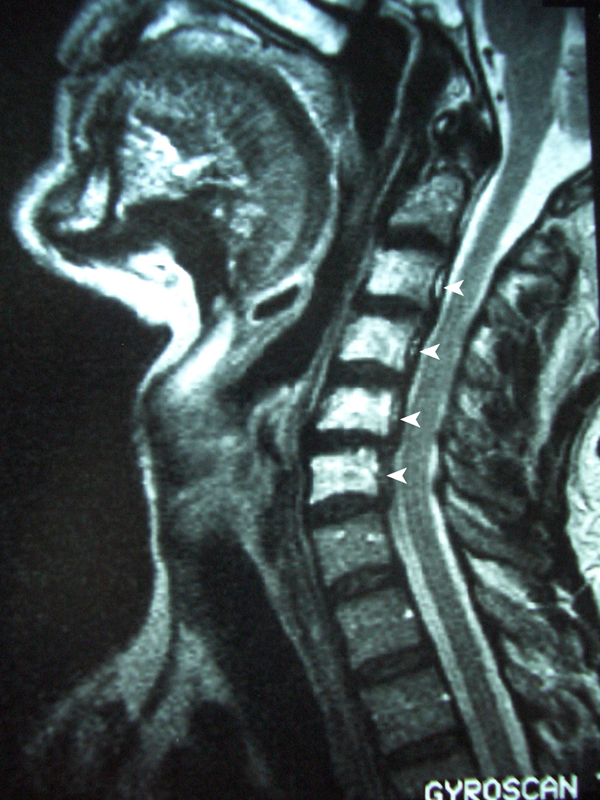
**December 1997: MRI scan of the neck showed marked narrowing of the airway**. An enhanced signal in C2 to C6 vertebral bodies (white arrows) and a loss of height in C5 were noted and deemed consistent with post-radiotherapy change.

At regular follow-up, there was no evidence of recurrence, but in December 1997, 15 years later, the patient was admitted as an emergency with sudden onset of stridor. Laryngoscopy revealed immobile bilateral vocal cords. An MRI scan of his neck showed marked narrowing of the airway. An enhanced signal in C2 to C6 vertebral bodies and a loss of height in C5 was noted and deemed consistent with post-radiotherapy change (see Figure 1). Due to the severity of the airway compromise, an urgent tracheostomy was performed.

In February 1998, microlaryngoscopy and laser right arytenoidectomy was performed to facilitate decannulation. In May 1998, a second tracheostomy was needed for airway difficulty, but successful decannulation was achieved. In July 1998, the stridor recurred. The only positive finding was fibrotic supraglottic stenosis. Several biopsies excluded recurrence of tumour as a cause.

The patient was keen to avoid a permanent tracheostomy as he was an enthusiastic windsurfer and wanted to continue his hobby. In January 1999, a formal partial vertical hemi-laryngectomy was performed. There was evidence of long-standing post-radiotherapy fibrosis, scarring and contraction.

During November 1999, the patient required a third elective tracheostomy post-hemi-laryngectomy. Due to ongoing aspiration, inability to decannulate and severe laryngeal scarring, a total laryngectomy was performed in February 2000. Histology showed no malignancy and the specimen showed changes consistent with post-radiotherapy change.

In April 2000, the patient was fitted with a Blomsinger valve, but the procedure was complicated by the development of cellulitis. He was treated with antibiotics but continued to suffer with pain, tenderness and dysphagia. A contrasted swallow study was reported as showing external constriction and the patient was referred to our gastroenterology department for flexible endoscopy and oesophageal dilatation. Two weeks after the oesophageal dilatation, he complained of persistent pain in his right shoulder and discomfort over the lower cervical spine. An MRI scan showed a mass originating from C4/C5 which extended into the pre-cervical space and posteriorly into the spinal canal causing cord compression. The possibility of a metastatic deposit in the C5 vertebral body was noted, but this did not fit in with the clinical picture. There was no neurological deficit at this time, and he was treated with analgesics.

**Figure 2:**
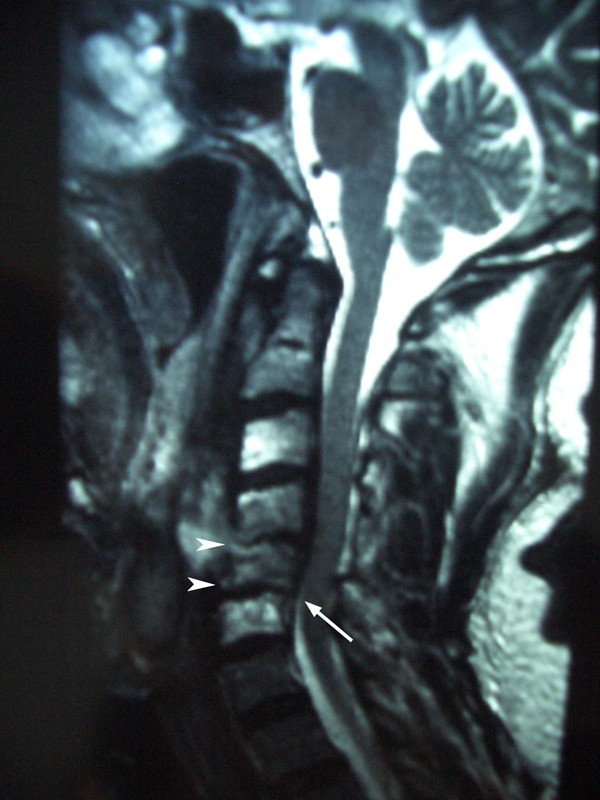
**June 2000: MRI scan revealed a reduction of the disc height at C4-5 and C5-6 (arrow heads), a loss of height of the C5 vertebral body and retrolisthesis of C5 on C6 (arrow)**.

He was admitted as an emergency 2 months later with increasing pain in his neck and, in addition, pain and paraesthesia in his right arm. A further MRI scan (see Figure 2) revealed a reduction of the disc height at C4-5 and C5-6, a loss of height of the C5 vertebral body and retrolisthesis of C5 on C6. Inflammatory markers were significantly elevated. The patient was immobilized in a Jerome Halo brace.

At surgery, there was no evidence of infection, and the prevertebral fascia was intact. The vertebral bodies of C4 and C5 were curetted and submitted for culture and histology. No infective organisms were found and no evidence of metastatic tumour was seen.

Two weeks later, the patient collapsed with severe pain, dizziness, paraesthesia in his legs and inability to walk. He was found to have signs of quadriplegia and a sensory deficit below C5 distribution. He was transferred to the neurosurgical unit where his neck was re-explored. Partial destruction of the bodies of C4 and C5 was noted and curettage of the vertebral bodies was performed. The posterior longitudinal ligament was incised and an epidural abscess was drained. No organisms were grown from these samples and only white cells were present. Biopsies again showed no evidence of tumour recurrence.

The patient was stabilized and transferred to a specialist spinal unit where he received intensive rehabilitation. He regained some power in both his upper and lower limbs, and is able to mobilize for short distances. He remains with an indwelling catheter, permanent tracheostome and is mostly wheelchair bound.

## Discussion

This case highlights a very serious but rare complication of radiation therapy to the larynx. In retrospect, the degeneration of C4/C5 vertebral bodies demonstrated on MRI and findings at operative exploration are thought to have been due to osteoradionecrosis of the cervical spine vertebrae secondary to the radiotherapy 20 years earlier and not due to metastatic disease as reported by the MRI scan.

A literature search using the Medline (1950 to 2006) and Embase (1975 to 2006) databases utilizing search terms "osteoradionecrosis", "cervical" and "avascular necrosis", yielded only one previous case report from North America relating to cervical osteoradionecrosis following radiotherapy for laryngeal cancer but involved extensive primary surgery and occurred 10 months after completion of radiotherapy [4].

This appears to be the first reported case where radiotherapy to the larynx was the sole cause of osteoradionecrosis of the cervical spine. Four other reports have been published describing osteoradionecrosis of the cervical spine following radiotherapy to head and neck neoplasms [4-6]. None of these were from a primary laryngeal carcinoma, although one was a patient with an unknown primary.

Laryngeal carcinoma is frequently treated with surgery and/or radiotherapy. By careful planning, vital structures such as the brain, spinal cord and eyes are protected from irradiation by manipulating the radiation field (wedges), doses and number of exposures [7]. Despite these precautions, there is a risk of radiation injury to soft tissue and bone. There are a plethora of literature/papers describing avascular necrosis to structures, such as the mandible, the temporal bones, the hyoid and thyroid, secondary to radiation osteoradionecrosis [8]-[12].

The patient presented in the case report received a total of 6600cGy of focused radiation therapy, 30 doses over 43 days for the treatment of a T1aN0M0 laryngeal carcinoma. Previous cases have been reported which describe the development of cervical osteoradionecrosis some 5 to 10 years after the initial radiation treatment [4]-[6]. These patients were all exposed to a cumulative dose of approximately 6000cGy. They presented with symptoms of neck pain and progressive quadriplegia, and these were initially thought to be due to either a recurrence of the treated tumour or a new primary.

Despite several hypotheses to address the pathophysiological process behind osteoradionecrosis, the hypothesis put forward by Marx in 1983 has been the most widely accepted. He postulates the combination of hypoxia, hypovascularity and hypocellularity, the 'three H' hypothesis, to describe post-radiation injury [3]. This proposes that the radiation-induced cellular injury and fibrosis renders the irradiated bone unable to increase its metabolic and nutritional requirements, and thus unable to replace the normal collagen and cellular components lost through routine wear and tear, resulting in tissue breakdown and necrosis. More recent papers suggest a fibro-atrophic mechanism over this traditional theory of vascular insufficiency [13].

Osteoradionecrosis usually presents within the first 12 to 24 months following radiation therapy, but it has been described to present up to 20 to 30 years after the initial radiation treatment [12]. The most common symptom of osteoradionecrosis is pain, but it may also present with a pathological fracture, or the formation of an abscess. Due to similarities in clinical presentation, it is vital that the possibility of tumour recurrence or a new primary cancer be excluded first.

The treatment of radiation necrosis has been described by a host of authors and includes hyperbaric oxygen, antibiotics, or debridement [7,9,12,13]. If diagnosed early, the condition may be treated successfully. Marx et al claimed a success rate of 95% in treating mandibular osteoradionecrosis [15]. However, the management of osteoradionecrosis remains controversial, and in particular, the efficacy of hyperbaric oxygen has been questioned [13,14].

## Conclusions

This is the first report of a patient with laryngeal cancer solely treated with radiotherapy who developed cervical osteoradionecrosis. The onset of osteoradionecrosis can be insidious and the location atypical. Radiotherapy carries a long-term risk of complications, including osteoradionecrosis which may present 20 years later with significant implications.

## Abbreviations

C2: second cervical vertebra; C5: fifth cervical vertebra; C6: sixth cervical vertebra; cGy: centigray; MRI: magnetic resonance imaging; T1aN0M0: clinical staging according to International Union Against Cancer: TNM Classification of Malignant Tumours, Sixth Edition.

## Consent

Written informed consent was obtained from the patient for publication of this case report and any accompanying images. A copy of the written consent is available for review by the Editor-in-Chief of this journal.

## Competing interests

The authors declare that they have no competing interests.

## Authors' contributions

FCVW and MS gathered background information from case notes, radiology reports and personal communication with the consultants involved, and carried out a review of the literature. RT conceived the study, and participated in its design and coordination and all of the authors helped to draft the manuscript. All authors read and approved the final manuscript.
